# Systematic review of the accuracy of plasma preparation tubes for HIV viral load testing

**DOI:** 10.1371/journal.pone.0225393

**Published:** 2019-11-21

**Authors:** Robert Luo, Jessica Markby, Jilian Sacks, Lara Vojnov

**Affiliations:** 1 World Health Organization, Geneva, Switzerland; 2 Clinton Health Access Initiative, Boston, Massachusetts, United States of America; AIDS Healthcare Foundation, UNITED STATES

## Abstract

Expanding access to HIV viral load testing is essential to improving the care and treatment of people living with HIV/AIDS and ending the AIDS epidemic. Though significant investments have been made in the past five years, many high burden, low resource countries continue to have viral load access rates below 50%. Plasma preparation tubes (PPTs) can simplify storage, transport, and preparation of plasma used for viral load testing. A systematic review was conducted to evaluate the accuracy of plasma preparation tubes for HIV viral load testing. Study results regarding the accuracy of PPT viral load measurements across various storage and transportation conditions were examined. The quality of evidence was evaluated using GRADE and QUADAS-2 criteria. The review identified 16 studies using PPTs with data from 6,141 individuals from 1995 to 2014. Overall the quality of evidence was rated as moderate, with unclear applicability for studies evaluating viral load assays that are no longer commercially available. Significantly elevated viral load results (>0.3 log copies/ml difference) have been observed with PPTs; however, when manufacturer handling instructions are followed, when plasma is aliquoted into a secondary tube, or when PPTs are centrifuged prior to testing, PPT results only differed from standard EDTA plasma testing using commercially available viral load assays by a range on average of -0.03 to +0.08 log copies/ml across studies. Although spuriously elevated viral load results have been observed with PPTs, following proper sample handing techniques have been shown to provide accurate results. PPTs, therefore, provide a high quality alternative specimen type for countries seeking solutions to infrastructure and specimen transportation challenges in an effort to scale-up viral load testing and achieve 90-90-90 targets.

## Introduction

Viral load testing is the preferred method for monitoring individuals on HIV antiretroviral treatment [1]. Expanding access to viral load is a priority for improving the care and treatment of persons with HIV/AIDS, particularly those living in resource limited settings, and is essential for meeting the UNAIDS 90-90-90 goals for ending the AIDS epidemic [2]. The primary and preferred sample type for viral load testing is plasma [3], which is prepared from whole blood collected via venipuncture. Often whole blood is collected into an EDTA (ethylenediaminetetraacetic acid anti-coagulant) tube (purple or lavender cap). Whole blood in EDTA tubes, however, must be transported and centrifuged into plasma within 6–24 hours of sample collection, depending on the manufacturer [4]. This can be restrictive for many countries and health care facilities. Though investments and viral load scale-up have been significant since 2013, access has been limited in high burden countries to those with advanced infrastructure and robust specimen transport networks [5,6]. Dried blood spots, which can be prepared from fingerstick whole blood, provide an alternative method for sample collection; however, limited progress has been made in ensuring the quality of using dried blood spots for HIV viral load testing through international regulatory approvals [7]. Because of this, countries have sought alternative specimen types and/or technologies to further support and expand viral load testing access.

Unlike standard EDTA blood collection tubes, plasma preparation tubes (PPTs) can facilitate simpler handling and storage of plasma for HIV viral load testing. PPTs use the same EDTA anticoagulant, but contain a gel that separates the plasma from blood cells during centrifugation [8]. After the blood is collected, the PPT is spun in a centrifuge within 24 hours and a gel barrier inside the PPT separates the plasma from the rest of the whole blood so the plasma can be used for HIV viral load testing. The same plasma specimen volume is used for the viral load assay; therefore, limits of detection are synonymous with EDTA plasma. Additionally, plasma can be stored in the PPTs *in situ* if desired, without the need for additional manual pipetting steps to a secondary tube, which can reduce the risk of sample handling errors and the potential for cross-contamination [9].

A recent systematic review on blood stability indicated that PPTs are likely stable for at least 5–7 days, far beyond manufacturer instructions, depending on storage temperatures [9]. However, despite their growing and widespread use in many countries, including high burden, low resource settings such as Kenya and South Africa, and their ability to increase access to viral load testing, there has not yet been a systematic review examining the accuracy of PPTs for HIV viral load testing. Additionally, conflicting results reported in the literature with regards to their accuracy and optimal handling methods have raised questions concerning their use with different manufacturers’ viral load assays. Therefore, a systematic review was conducted to evaluate the accuracy of PPTs for HIV viral load testing.

## Methods

A systematic review was performed according to the Preferred Reporting Items for Systematic Reviews and Meta-Analyses (PRISMA, completed checklist in S1 Table) [10] following a pre-defined study protocol. Three databases [PubMed/Medline, International AIDS Society conference proceedings, and CROI (Conference on Retroviruses and Opportunistic Infections) conference proceedings] were searched using search terms from the protocol (detailed in the S1 File) in January 2019 to identify potentially relevant studies. No restrictions on publication year, publication status, or language were used.

Two reviewers independently screened all titles and abstracts for inclusion and reviewed all potentially relevant studies in full. Studies were included if they evaluated the accuracy of PPTs for HIV viral load testing by comparing viral load results from PPTs to viral load results from either EDTA tubes or PPT tubes tested on another viral load assay approved by a stringent regulatory authority. Studies were excluded if they did not include a proper comparator or if they were purely review articles.

Data were extracted and summarized for all included studies, outlining the study design, methods, and principle components of each study (e.g., sample size, viral load assay used, storage and transport conditions of samples). The primary outcome assessed was accuracy of the PPT measurements, defined by studies as a significant statistical or clinical difference between the PPT viral load measurements and the comparator viral load result used in the study. The quality and risk of bias of individual studies were assessed using QUADAS-2 criteria [11], and the entire body of evidence included in the review was assessed using GRADE criteria [12].

## Results

**Fig 1:**
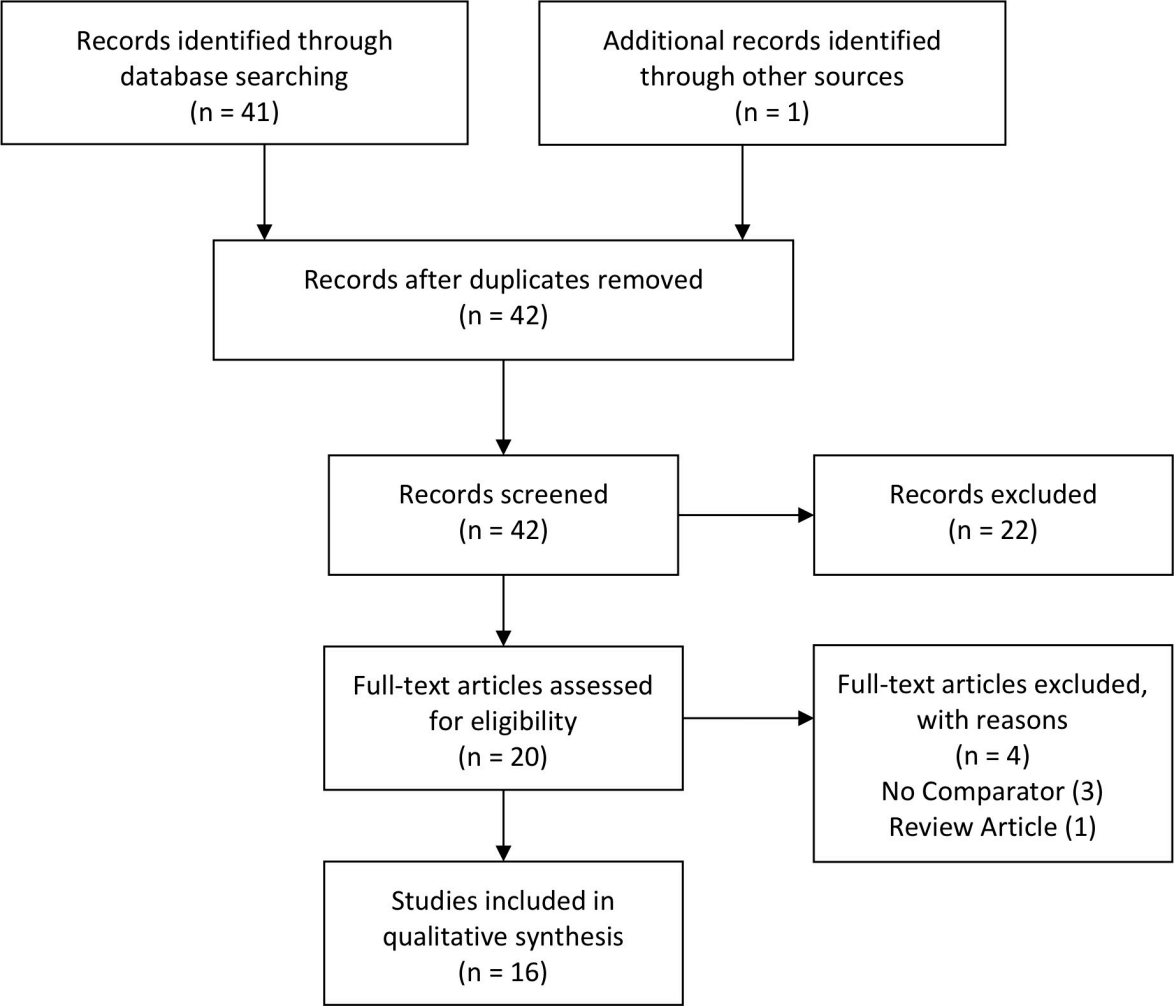
PRISMA diagram of studies.

The review identified forty-two studies through database searches, of which sixteen met the inclusion criteria (Fig 1). Overall the studies were published between 1995–2014 and included samples from 6,141 individuals. Although the majority of studies (11/16, 69%) were conducted in the United States, the majority of samples (4049/6141, 66%) included came from a large retrospective study done in Norway. Due to the vast difference in sample storage and transport conditions among studies, the results were only qualitatively summarized, and a meta-analysis was not undertaken. The main characteristics for each study are summarized in Table 1.

**Table 1:** Summary of Studies.

Author / Year	Country	Number of Subjects	Viral Load Test(s) Used	Storage/Transport Conditions	PPT Impact on Viral Load Results
Holodniy 1995	US	40	Chiron Quantiplex HIV RNA	Storage at RT for 2h, 6h and 30h, frozen as aliquots at -70°C	Significant decrease (33%) only if whole blood stored for 30h
Ginocchio 1997	US	10	Organon Teknika NASBA HIV-1 RNA QT Assay	Centrifuged after 2h, 8h, and 30h at 4°C and 23°C, aliquoting/freezing at -70°C	No significant differences, mean variations all within ±0.3 log cp/ml
Holodniy 2000	US	19	Roche Amplicor HIV-1 v1.0	Storage at -70°C, 4°C, and RT, frozen *in situ* and as aliquots	No significant differences, p<0.05 for all comparisons
Elbeik 2005	US	159	Bayer VERSANT HIV-1 RNA 3.0	RT for 4h, 24h, and 72h, then frozen *in situ*	No significant decrease in viral load after 24h (13% decrease), but 37% decrease after 72h
Giordano 2006	Multiple	584	Roche Amplicor HIV-1 v1.0 and v1.5	Centrifuged within 6h, frozen *in situ* at -70°C 3–6 months	11% increase in detectable viral loads at 400 cp/ml, 34% increase for in detectable viral loads at 50 cp/ml
Griffith 2006	US	112	Roche Amplicor HIV-1 v1.5	Centrifuged within 2h, frozen *in situ*	PPTs higher at low viral loads, with 40% detectable in PPTs when undetectable in EDTA
Wan 2010	US	50	Roche Amplicor HIV-1 v1.5	Centrifuged within 2h at RT, frozen *in situ* at -70°C	Significant increase (p = 0.002) with 28% detectable viral loads in PPTs when EDTA undetectable
Salimnia 2005	US	13	Roche Amplicor HIV-1 v1.5	RT for 4h, then frozen *in situ* or in aliquots	Concordant results for aliquots, 62% discordant (>0.5 log cp/ml higher) if frozen *in situ*
Garcia-Bujalance 2007	Spain	51	Roche Amplicor HIV-1 v1.5	Centrifuged within 2h, frozen *in situ* or in aliquots at -70°C	Higher viral loads (mean 0.6 log cp/ml higher) if frozen *in situ*
Rebeiro 2008	US	252	Roche Amplicor HIV-1 v1.5, Roche CAP/CTM v1.0	Frozen in aliquots or *in situ*	57% with >0.5 log cp/ml increase if frozen *in situ* instead of in aliquots
Kran 2009	Norway	4049	Roche CAP/CTM v1.0	Transport 2h, with centrifugation before or after transport, frozen as aliquots at -70°C	37% more detectable viral loads if centrifuged before transport instead of after
Kraft 2013	US	63	Roche CAP/CTM v1.0	Aliquoted before and/or after transport, stored at 4°C and -20°C	No significant differences if centrifuged prior to testing (correlation >0.80), but 35% with detectable viral load if centrifuged before transport and not after
Fernandes 2010	US	64	Abbott RealTi*m*e HIV-1	Transport 2h-6h, frozen *in situ* or as aliquots at -70°C	No significant differences, r^2^ > 0.92 for all comparisons
Adachi 2014	Canada	65	Abbott RealTi*m*e HIV-1, Roche CAP/CTM v2.0,	Routine testing, transport parameters not described	No significant differences, Roche tended to have higher viral loads when EDTA viral load < 200 cp/ml
Cloherty 2014	US	349	Abbott RealTi*m*e HIV-1, Roche CAP/CTM v2.0	Transport at RT < 4h, frozen *in situ* at -20°C	Roche viral loads higher than Abbott; 56% of samples with detectable viral load on Roche but undetectable on Abbott
Goedhals 2013	South Africa	261	Roche CAP/CTM v2.0	Transport at RT < 8h, frozen as aliquots at -80°C	No significant differences (mean 0.04 log cp/ml)

PPT, plasma preparation tube; US, United States; RT, room temperature; h, hours; cp/ml, copies/milliliter; CAP/CTM, COBAS AmpliPrep/COBAS TaqMan; v, version; EDTA, standard blood tube without gel separator

The quality of studies included in the systematic review was moderate overall. Evaluation of each study following QUADAS-2 criteria showed a low risk of bias. Samples were taken from patients with a wide range of HIV viral loads. Of note, the majority of studies (14/16, 88%) were either done in North America or Western Europe, with only two of the studies including samples from high burden HIV regions. However, that did not affect the risk of bias, as studies evaluated a broad range of sample handling conditions, including transportation from remote clinics. A majority of studies (12/16, 75%) also had unclear applicability for the index and reference tests used, since the tests were no longer commercially available and have been replaced by newer tests (detailed in S2 Table). GRADE assessment did not reveal any serious risk of bias, indirectness or imprecision, although inconsistency was high across studies due to the variability in storage and transportation conditions (Table 2).

**Table 2:** GRADE evaluation of evidence quality.

Number of Studies	Study Design	Risk of bias	Inconsistency	Indirectness	Imprecision	Quality	Importance
16	Cohort Studies	Not Serious	Serious^1^	Not Serious	Not Serious	Moderate	Critical

^1^Studies varied widely in storage and transportation conditions of samples prior to testing.

The earliest group of studies were published between 1995 and 2005 and validated that PPTs could be used with no significant differences in viral load results [13–16]. However, later studies demonstrated elevated viral loads using PPTs (over a 0.3 log copies/ml increase compared to standard EDTA plasma), particularly at viral loads less than 5,000 copies/ml [17–19]. The elevated viral load results were likely due to the leakage of cells containing HIV nucleic acids, such as proviral HIV DNA and intracellular RNA, from the cellular component of whole blood, that moved back through the gel barrier into the plasma. Additional studies found this issue could be resolved by either aliquoting the plasma into a secondary tube after the initial centrifugation [20–22], or repeating centrifugation after transport of the PPTs to the lab prior to aliquoting and testing [23–24].

Four published studies evaluated PPTs on viral load assays that were commercially available at the time of this review (Abbott RealTi*m*e HIV-1 and Roche COBAS AmpliPREP/COBAS TaqMan HIV-1 Test, v2.0) [25–28]. The three studies evaluating the Abbott viral load assay showed no significant change in viral load results regardless of if the PPTs were frozen and thawed or transported after initial centrifugation and prior to testing [25–27]. The three studies assessing the Roche assay found elevated viral load results if the PPTs were frozen or transported without a second centrifugation prior to testing [26–28]. These viral load results were found to be up to 5,260 copies/ml higher than plasma prepared from a standard EDTA collection tube, with the differences being most noticeable for plasma viral loads less than 1,000 copies/ml due to their relative magnitude compared to the standard EDTA viral load result. Consequently, manufacturer instructions recommend an additional centrifugation step prior to testing using the Roche assay. For both the Abbott and Roche assays, aliquoting the plasma into a secondary tube after initial centrifugation also ensured accurate viral load results. Table 3 summarizes the methods published in the literature that support accurate results for the current use of PPTs. When proper handling methods were followed for each assay, the mean viral load results from PPTs evaluated in the studies only differed by a range of -0.03 to +0.08 log copies/ml from the mean viral load results from standard EDTA plasma. This difference is well-within the standard variation seen in viral load assays even when the same sample is tested twice, and far below the 0.3–0.5 log copies/ml difference that is considered clinically significant [29,30].

**Table 3:** Published handling methods for commercially available PPTs and viral load assays.

Product	Published PPT handling methods providing accurate viral load results
Abbott RealTi*m*e HIV-1 [25–27]	• Aliquoting plasma into new tube after initial centrifugation • Freezing PPT at -20°C after initial centrifugation and thawing prior to testing, without the necessity for another centrifugation step • Transporting PPT after initial centrifugation between sites prior to testing, without the necessity for another centrifugation step
Roche COBAS^®^ AmpliPREP/COBAS^®^ TaqMan^®^ HIV-1 Test, v2.0 [26–28]	• Aliquoting plasma into new tube after initial centrifugation • Repeat centrifugation after transport or freezing of PPT in order to ensure complete separation of the cellular and plasma components of blood prior to testing • Note: In the absence of repeat centrifugation after freezing and thawing of PPTs or after transport of PPTs, some viral load results were observed to be erroneously high. Repeat centrifugation is not necessary if the plasma has already been aliquoted into a new tube prior to freezing or transport.
BD Vacutainer^®^ PPT^TM^ [8]	• Centrifuge for at least 10 minutes at 1100 x g at room temperature, within 6 hours of collecting whole blood to prepare plasma • Follow assay manufacturer instructions for storage and transport: typically PPTs can be stored at ambient temperature for 1 day or refrigerated at 4°C for up to 5 days; if longer storage is desired, the plasma should be frozen

PPT, plasma preparation tube; BD, Becton-Dickinson

## Discussion

PPTs allow plasma to be prepared, stored, and transported in the same tube used to collect venous whole blood. PPTs have been shown to provide equivalent viral load results to plasma from standard EDTA tubes if proper handling of PPTs is followed according to the guidance from independently published studies included in this review as well as manufacturer instructions [31,32].

Regardless of assay type used, centrifugation of PPTs and/or aliquoting of plasma into a secondary tube prior to viral load testing has also been consistently shown to prevent falsely elevated viral load results. Performing these steps will ensure that the cellular component of whole blood has been completely separated from the plasma in order to prevent HIV proviral DNA, intracellular HIV RNA, and HIV particles associated with platelets from falsely elevating the viral load results [33,34]. Assays that are more HIV RNA specific are likely to be less impacted by the presence of proviral DNA and may explain why some assays still show accurate viral load results even when PPTs are frozen and thawed *in situ*, without a separate aliquoting or centrifugation step.

PPTs can improve access to viral load testing in several ways. The ability to store plasma for longer periods of time than in standard EDTA tubes, as indicated by an earlier systematic review [9], could expand the reach of viral load laboratories, which is particularly useful in resource limited settings. Additionally, easier sample handling with fewer manual steps and a reduced risk of sample contamination if the plasma is kept in the PPT can streamline laboratory workflows and increase testing throughput. Although they are typically more expensive than EDTA tubes, if PPTs are available and staff can be trained on their use, they provide another option for HIV programs deciding how to expand coverage of viral load testing. Laboratories using PPTs must then be familiar with proper handling methods and any additional equipment (e.g. secondary tubes for aliquoting, centrifugation needs prior to testing) needed to ensure accurate results. Existing procurement and supply chain mechanisms which have simplified purchasing of phlebotomy supplies for many countries through all-in-one sample bundles would also need to be updated to include PPTs, since they typically only include EDTA tubes currently. Table 4 summarizes the advantages and challenges associated with using PPTs for viral load testing identified through this review.

**Table 4:** Advantages and challenges associated with PPTs.

Advantages	Challenges
• Fewer manual sampling handling steps than standard EDTA tubes • Reduced risk of sample contamination and laboratory errors if plasma is not aliquoted into a new tube • Ability to store plasma for longer periods of time than uncentrifuged whole blood, which can facilitate longer transport times to the laboratory	• Higher cost of PPTs compared to standard EDTA tubes • Programmatic complexities involving supply chain logistics, staff training, and proper implementation of PPTs • Centrifuges are required on-site for immediate plasma separation • Primary tube sampling not possible if assay manufacturer-specific instructions are not followed, e.g. a repeat centrifugation step may be necessary prior to testing • Existing sample bundles (phlebotomy kits) used in many countries typically only include EDTA tubes

PPT, plasma preparation tube; EDTA, standard blood tube without gel separator

A major limitation of this review is that it does not encompass all PPTs and viral load tests that are currently commercially available. All of the studies in this review evaluated only BD Vacutainer^®^ PPT^TM^s, the most commonly used PPT brand. Other PPTs, also referred to as EDTA with gel separator tubes, are commercially available from manufacturers such as Grenier and TUD [35,36]. Additionally, other viral load assays such as the Cepheid Xpert HIV-1 and Hologic Aptima HIV-1 Quant Dx assays are the only other tests, in addition to the Abbott and Roche viral load assays, that are currently commercially available with regulatory approval for the use of PPTs; however, their instructions for use do not provide any further specific guidance on how PPTs should be used [37,38]. No independent studies were found evaluating other brands of PPTs or the use of PPTs with these viral load assays.

While the majority of studies were done in North America or Western Europe, the studies still examined a wide range of storage and transport conditions that could be found anywhere in the world indicating generalizability of the results. Additionally, when PPTs were handled properly, the results were accurate regardless of the population in which the study was conducted. Scientifically, PPTs would not be expected to separate plasma differently in viral load laboratories in high burden HIV countries, and their gel separation capability would not be expected to be affected by any known patient characteristics. However, it is important to note that methods and storage/shipping times were simulated in the laboratory, rather than studied in decentralized settings. Additionally, further research on other brands of PPTs as well as with other viral load assays that have regulatory approval would be helpful to confirm the findings of the studies in this review.

Overall, this review summarized results from 16 studies of PPTs involving results from over 6,000 individuals. Consideration of PPTs may be particularly worthwhile in settings where simpler plasma preparation, reduced cross-contamination risk, and longer sample transport times can facilitate the scale up of viral load testing. PPTs will add to the menu of options available to countries to expand access to viral load, particularly in settings that may require more support and extended shipping or storage times compared to standard EDTA samples. Increasing access to viral load will improve patient care and services, while also supporting country goals to reach the 90-90-90 targets.

## Supporting information

S1 File Search terms protocol. (DOCX)

S1 Table PRISMA checklist. (DOCX)

S2 Table QUADAS-2 assessment of individual studies. (DOCX)
