# Supplementary material for: Systematic review of the accuracy of plasma preparation tubes for HIV viral load testing
Source: PLoS One. 2019 Nov 21;14(11):e0225393. doi: 10.1371/journal.pone.0225393 (PMC6874077; doi:10.1371/journal.pone.0225393)
Supplement: S1 File — (DOCX) [file pone.0225393.s001.docx]

**S1 File. Search Terms Protocol**

(HIV OR HIV infections OR hiv OR hiv-1* OR hiv-2* OR hiv1 OR hiv2 OR hiv infect* OR human immunodeficiency virus OR human immunodeficiency virus OR human immuno-deficiency virus OR human immune-deficiency virus OR ((human immun*) AND (deficiency virus)) OR acquired immunodeficiency syndrome OR acquired immunodeficiency syndrome OR acquired immuno-deficiency syndrome OR acquired immune-deficiency syndrome OR ((acquired immun*) AND (deficiency syndrome)) OR sexually transmitted diseases, viral or Human T Cell Lymphotropic Virus* or Human T Lymphotropic Virus* or Human T Cell Leukemia Virus* or LAV HTLV III or Lymphadenopathy Associated Virus* or HIV or HIV 1 or HIV 2 or HIV/AIDS or HIV I or LAV 2 or LAV HTLV III or HIV II or HTLV III or HTLV IV or SBL 6669 or AIDS)

AND

(Viral Load or Virus Load* or Viral Burden* or Virus Burden* or Virus Titer* or Viral Titer* or VL or VLs or RNA level or RNA level*)

AND

(plasma preparation tube* or ppt* or vacutainer ppt*)
