# Supplementary material for: Systematic review of the accuracy of plasma preparation tubes for HIV viral load testing
Source: PLoS One. 2019 Nov 21;14(11):e0225393. doi: 10.1371/journal.pone.0225393 (PMC6874077; doi:10.1371/journal.pone.0225393)
Supplement: S2 Table — (DOCX) [file pone.0225393.s003.docx]

**S2 Table. QUADAS-2 assessment of individual studies**

|  | Risk of Bias | | | | Applicability Concerns | | |
| --- | --- | --- | --- | --- | --- | --- | --- |
| Study | Patient Selection | Index Test | Reference Standard | Flow &  Timing | Patient Selection | Index Test | Reference Standard |
| Holodniy 1995 | Low | Low | Low | Low | Low | Unclear | Unclear |
| Ginocchio 1997 | Low | Low | Low | Low | Low | Unclear | Unclear |
| Holodniy 2000 | Low | Low | Low | Low | Low | Unclear | Unclear |
| Elbeik 2005 | Low | Low | Low | Low | Low | Unclear | Unclear |
| Giordano 2006 | Low | Low | Low | Low | Low | Unclear | Unclear |
| Griffith 2006 | Low | Low | Low | Low | Low | Unclear | Unclear |
| Wan 2010 | Low | Low | Low | Low | Low | Unclear | Unclear |
| Salimnia 2005 | Low | Low | Low | Low | Low | Unclear | Unclear |
| Garcia-Bujalance 2007 | Low | Low | Low | Low | Low | Unclear | Unclear |
| Rebeiro 2008 | Low | Low | Low | Low | Low | Unclear | Unclear |
| Kran 2009 | Low | Low | Low | Low | Low | Unclear | Unclear |
| Kraft 2013 | Low | Low | Low | Low | Low | Unclear | Unclear |
| Fernandes 2010 | Low | Low | Low | Low | Low | Low | Low |
| Adachi 2014 | Low | Low | Low | Low | Low | Low | Low |
| Cloherty 2014 | Low | Low | Low | Low | Low | Low | Low |
| Goedhals 2013 | Low | Low | Low | Low | Low | Low | Low |
